# One-Step Transfer of Symmetric and Asymmetric Contacts for Large-Scale 2D Electronics and Optoelectronics

**DOI:** 10.1021/acsnano.5c09815

**Published:** 2025-07-23

**Authors:** Jingying Liu, Kaijian Xing, Lintao Li, Weiyao Zhao, Alastair Stacey, Islay Robertson, David A. Broadway, Jean-Philippe Tetienne, Dong-Chen Qi, Michael S. Fuhrer, Yufeng Hao, Qingdong Ou

**Affiliations:** † 58816 Macau University of Science and Technology Zhuhai MUST Science and Technology Research Institute, Zhuhai 519031, China; ‡ Macao Institute of Materials Science and Engineering (MIMSE), Faculty of Innovation Engineering, Macau University of Science and Technology, Taipa, Macao 999078, China; § School of Physics and Astronomy, 2541 Monash University, Clayton, Victoria 3800, Australia; ∥ National Laboratory of Solid State Microstructures, College of Engineering and Applied Sciences, Jiangsu Key Laboratory of Artificial Functional Materials and Collaborative Innovation Center of Advanced Microstructures, Nanjing University, Nanjing 210023, China; ⊥ Department of Materials Science & Engineering, Monash University, Clayton, Victoria 3800, Australia; # School of Science, 5376 RMIT University, Melbourne, Victoria 3000, Australia; ¶ Princeton Plasma Physics Laboratory, 100 Stellarator Road, Princeton, New Jersey 08540, United States; ∇ Centre for Materials Science, School of Chemistry and Physics, 1969 Queensland University of Technology, Brisbane, Queensland 4001, Australia

**Keywords:** electrode transfer, asymmetric contact, 2D electronics, 2D optoelectronics, wafer scale

## Abstract

Two-dimensional (2D) semiconductors are highly promising candidates for thin-film transistor applications due to their scalability, transferability, atomic thickness, and relatively high carrier mobility. However, a substantial performance gap remains between individual devices based on single-crystalline 2D films and wafer-scale integrated circuits, primarily due to defects introduced during conventional fabrication processes. Here, we report a diamond-assisted electrode transfer technique for the van der Waals integration of wafer-scale prefabricated electrode arrays onto 2D materials, enabling scalable electronics and optoelectronics. Implemented on metal–organic chemical vapor deposition-grown monolayer molybdenum disulfide, this method forms ultraclean metal–semiconductor interfaces, yielding field-effect transistors with excellent ohmic contacts, a low contact resistance of 400 Ω·μm, and a Schottky barrier height of only 9 meV. Furthermore, we demonstrate a scalable transistor array on monolayer molybdenum disulfide with excellent device performance uniformity, achieving an average field-effect mobility of 30 cm^2^ V^–1^ s^–1^ and an on/off current ratio exceeding 10^5^. Additionally, high photocurrent and responsivity were demonstrated in the array devices, showing their potential for excellent image detection. We further demonstrate the versatility of this technique by fabricating a Schottky diode array through a single-step transfer of asymmetric electrodes—low work function aluminum and high work function gold—onto monolayer tungsten diselenide. This approach provides a clean, effective solution for contact engineering in 2D materials, offering a viable pathway toward wafer-scale, high-performance 2D electronics, optoelectronics, and integrated circuits.

## Graphical abstract

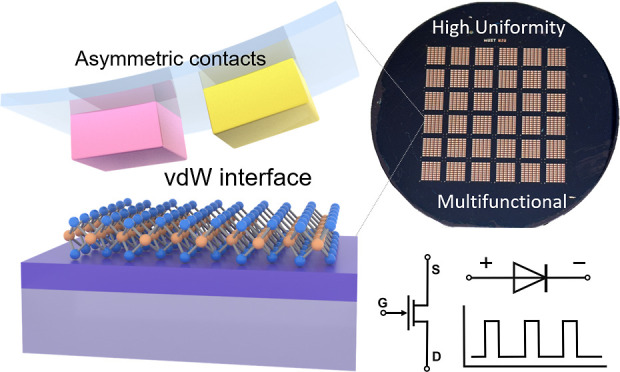

## 1 Introduction

Two-dimensional (2D) semiconductors have been intensively investigated for novel device applications due to their atomically thin nature, dangling-bond-free surfaces, and so on. In particular, transition metal dichalcogenides (TMDs), with a band gap of approximately 1.5 eV, are promising candidates for field-effect transistors (FET),^1^ micro/nano-light-emitting diodes,^2^ and photodetectors.^3^ However, the realization of practical industrial applications requires the achievement of wafer-scale, uniform, and high-quality single-crystalline 2D TMDs, which remains a significant challenge. As an alternative, atomically thin polycrystalline TMDs offer a viable pathway for enabling wafer-scale device applications.^4,5^ Among the various strategies explored for the large-scale synthesis of polycrystalline TMDs, chemical vapor deposition (CVD)^6^ and metal–organic chemical vapor deposition (MOCVD)^7^ have been the most exploited and versatile. Notably, MOCVD exhibits superior performance in terms of uniformity, compositional control, and repeatability, attributable to its use of gaseous precursors and precise control over growth parameters.

However, poor metal–TMD interfaces degrade contact performance and hinder the large-scale implementation of 2D electronics based on polycrystalline TMD film.^8,9^ The conventional photolithography and high-energy deposition damaged the atomically thin films, leading to additional defects, metal diffusion, and undesirable chemical bonding.^10^ These issues contribute to strong Fermi-level pinning (FLP), resulting in unpredictable Schottky barrier heights (SBH) and high contact resistance.^11^ The uncontrollable high contact resistance often leads to significant device-to-device variation and poor overall device performance, thereby constraining the development of practical electronics and optoelectronics that demand high uniformity and batch-to-batch repeatability.^12–14^

To optimize the metal-2D semiconductor interfaces, state-of-the-art contact technologies, such as semimetal contacts,^15–18^ charge transfer contacts,^19^ 2D/3D hybrid stack fabrication process^20–22^ and the use of sacrificed buffer layers,^23,24^ have been explored. Additionally, the physical transfer of prepatterned metal contacts onto 2D materials has emerged as a simple way to form the near-ideal van der Waals (vdW) interface between metals and 2D materials.^25–32^ However, these strategies have been mostly focused on single-crystalline TMD flakes, while the junction between large-area polycrystalline TMDs and metals remains less studied. Although recent works have demonstrated vdW contact integration on CVD-based TMDs,^33,34^ these efforts have mainly focused on high-work-function metal, such as Au, which are relatively easy to transfer. In contrast, certain industry-preferred metals with strong adhesion to substrate cannot be picked up alongside Au in a single step, thereby limiting the reliable transfer of large-area asymmetric contacts. Consequently, there is a critical need to develop a scalable transfer technique capable of accommodating arbitrary metals at a large scale for MOCVD-based TMDs.

Here, we developed a reliable electrode pick-up-and-place technique for scalable 2D array electronics and optoelectronics based on polycrystalline TMDs by using a recently developed diamond-assisted metal transfer strategy.^32^ Table S1 highlights the advantages of this diamond-assisted transfer technique, particularly its scalability in asymmetric contact array at the wafer scale. This method also eliminates the need for a sacrificial layer, enables substrate reusability, and is compatible with the standard photolithography process, offering significant benefit over other reported transfer techniques. FET array devices based on MOCVD-grown MoS_2_ with transferred symmetric vdW electrodes (Au) exhibit nearly ideal ohmic contacts, achieving low contact resistance of 400 Ω·μm at room temperature, consistent with the low extracted SBH of 9 meV. The achievement of low resistance and the ohmic nature of the contact enable the exploration of the intrinsic properties of MOCVD-grown monolayer MoS_2_ as a channel material, revealing a metal–insulator phase transition at low temperature (10 K) and saturated mobility of 104 cm^2^ V^–1^ s^–1^ at 77 K. In addition, the FET arrays demonstrate a 100% device yield and high uniformity of device performance, achieving average mobility of 30 cm^2^ V^–1^ s^–1^ (300 K), and on/off ratio ∼10^6^, respectively. Furthermore, we demonstrated the one-step formation of scalable asymmetric contacts for a MOCVD-grown WSe_2_ Schottky diode array. For optoelectronic applications, the MoS_2_-based photodetectors presented uniformity in photo response to white light and image-capture capability across this 2D array. We also demonstrate a superior photoresponsivity of phototransistors with vdW electrodes compared to the ones with evaporated electrodes. Overall, the transferred large-area top metal contacts provide a pathway to high-performance, wafer-scale electronics based on MOCVD-grown polycrystalline TMDs and highlight the potential as a key enabler for the future commercialization of MOCVD-grown TMD technologies.

## 2 Results and Discussion

### 2.1 Electrical Characterization of Single FET

For device fabrication, we employed the diamond-assisted transfer technology to integrate both large-scale symmetric contacts and asymmetric contacts on 2D TMDs toward high-performance electronics and optoelectronics. Highly crystalline monolayer MoS_2_ and WSe_2_ synthesized by MOCVD (Figure S1) were used as representative TMD films to ensure uniform continuous film across a large area.^35,36^ TEM analysis in Figure S1f–j displays a continuous monolayer film, indicating high crystallinity of the MoS_2_ film. To accomplish the diamond-assisted contact transfer, first, a polished diamond surface was hydrogenated to form a low-energy surface (details in method), and metal electrodes (i.e., Au–Au pair, Al–Au pair) were deposited onto the hydrogenated surface using a standard photolithography process.^37^ The metal contact arrays were then smoothly peeled off and laminated on MOCVD TMD films by a dry transfer method (Figure 1a), which minimized the contamination at the metal–semiconductor interface, such as photoresist residual. In addition, nearly 100% of the metal contacts maintained their original geometries without any cleavage or folding after landing on the TMD film (Figure S2), demonstrating the reliability of this transfer technique. Finally, the array of active channels was defined and patterned through reactive ion etching, ensuring high-quality vdW interfaces between metal contacts and TMD semiconductors. Furthermore, we also demonstrated that this strategy was scalable to wafer-scale device fabrication. As illustrated in Figure 1b, multiple asymmetric electrode arrays can be integrated on the wafer-level (two in.) MOCVD-grown WSe_2_.

**Figure 1:**
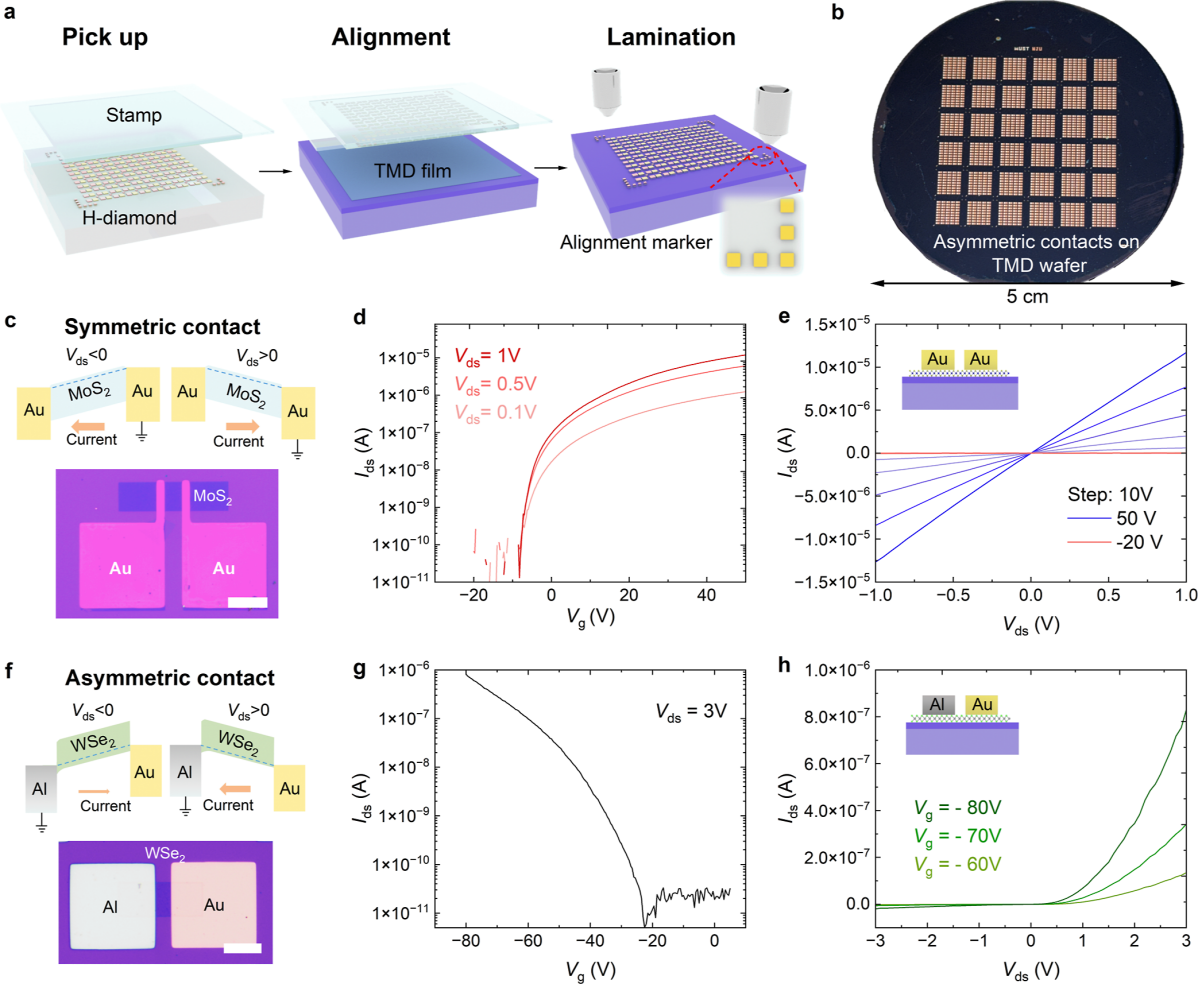
Fabrication process and transport characterizations of the field-effect transistor (FET) array using transferred vdW contacts. (a) Schematic of the array device fabrication using vdW contacts by diamond-assisted electrode transfer technology. (b) Photography of asymmetric contact arrays (Au and Al/Pd) transferred on the WSe_2_ film on a 2-in. wafer. (c) Band diagrams and optical image of TMD transistors with transferred symmetric Au contacts. Scale bar, 30 μm. (d,e) Transfer and output curves of a randomly selected MoS_2_ FET with vdW Au contacts. The inset of (e) is the schematic of the transistor. (f) Band diagram and optical image of a WSe_2_ Schottky diode with transferred asymmetric Au and Al/Pd contacts. Scale bar, 30 μm. (g,h) Transfer and output curves of a typical WSe_2_ Schottky diode. The inset of (h) is the schematic of the diode.

Before systematically characterizing the array devices (MoS_2_ with symmetric contacts and WSe_2_ with asymmetric contacts), we first studied the performance of a single device from each array. The randomly selected MoS_2_ FET showed typical *n*-type conductivity under static electric field-effect doping with an on/off ratio of ∼10^6^ (notably limited by the instrument resolution), as shown in Figure 1d. The linear current–voltage curves at different gate biases suggest ohmic behavior and negligible Schottky barriers at both Au–MoS_2_ interfaces (Figure 1e). In addition, the source-drain current of MoS_2_ with vdW Au contacts is more than an order of magnitude higher than that of MoS_2_ with evaporated contacts at the same *V*_ds_ and *V*_g_, suggesting superior contact performance for vdW electrodes. The transport data are consistent with the band diagram in Figure 1c, indicating a low Schottky barrier height (SBH) between transferred Au and polycrystalline monolayer MoS_2_.

Since a near-ideal interface can be formed between the vdW-integrated 3D metal contact and 2D semiconductor materials, both ohmic and Schottky contacts can be obtained by tuning the SBH with different metal electrodes of varying work functions.^25^ To illustrate this, we demonstrate a monolayer WSe_2_ device with transferred asymmetric metal electrodes Al (forming a Schottky contact) and Au (forming an ohmic contact). The Al electrode was connected to the electrical ground, while the source-drain voltage was applied to the Au electrode (Figure 1f). Figure 1g shows the transfer curve of the device, demonstrating typical hole conduction behavior at negative gate bias, characteristic of a WSe_2_-based Schottky diode. The output curves (Figure 1h) illustrate pronounced rectification behavior, which depends on the gate voltage ranging from −60 to −80 V. The nonlinear drain current-drain voltage relationship and relatively high rectification ratio ∼10^2^ indicate a relatively large SBH at the Al/WSe_2_ interface and a low SBH at the Au/WSe_2_ interface.

To quantify the SBH between Au and the MOCVD-grown MoS_2_ monolayer, we characterized the electrical transport properties of the Au/MoS_2_ FET at different temperatures. The *I*_ds_ at 77 K exceeded that at 300 K when the gate bias was larger than ∼60 V (Figure 2a). At the cryogenic regime (10 K), *I*_ds_ surpassed its 300 K value when the gate bias was over 100 V (Figure S3), suggesting a metal–insulator transition in this device. This indicates that the channel resistance dominated the overall device resistance rather than the contact resistance, implying that the contact resistance was minimal. The output curves in Figure 2b, measured at 77 K under different gate biases, display slight nonlinearity in the low *V*_ds_ region (−0.2 to 0.2 V), which can be attributed to the presence of a small Schottky barrier between Au and MoS_2_. The two-terminal field-effect mobility increased from 30 cm^2^ V^–1^ s^–1^ at 300 K to 104 cm^2^ V^–1^ s^–1^ at 77 K before reaching saturation (Figure 2c). From 175 to 300 K, we fit the generic temperature dependence of field-effect mobility, μ ∼ *T* ^–γ^. The value of γ was extracted to be ∼1.7, which is in good agreement with the theoretical prediction for monolayer MoS_2_.^38^ Below 77 K, mobility only experienced a slight decrease from 104 cm^2^ V^–1^ s^–1^ to 94 cm^2^ V^–1^ s^–1^, which might be due to the small SBH at the interface of metal/TMD. Further analysis using Arrhenius plots (Figure 2d) enabled a quantitative estimation of the SBH for the Au/MoS_2_ interface. As shown in Figure 2e, the SBH was extracted from the flat-band condition, corresponding to the onset of a deviation from the linear region. The Au/MoS_2_ interface exhibited an exceptionally low SBH of 9 meV, significantly lower than values reported in previous studies.^39,40^

**Figure 2:**
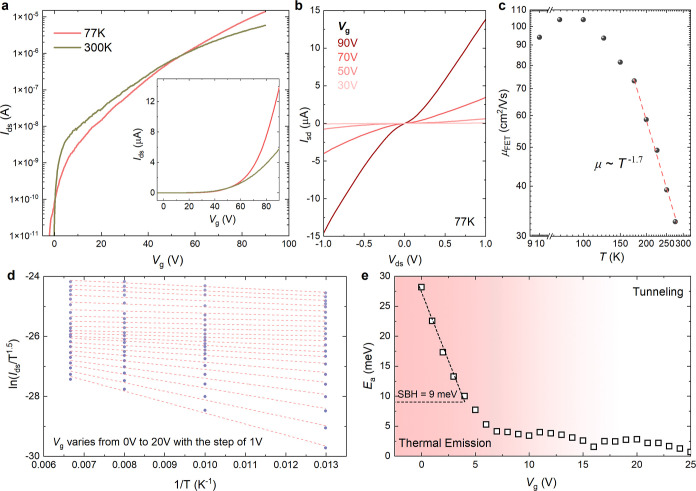
Temperature-dependent transport properties and Schottky barrier height analysis of monolayer MoS_2_ FETs with vdW Au electrodes. (a) Transfer characteristics of the devices at different temperatures (77 K and 300 K). (b) Output curves at 77 K for different back-gate voltages (*V*_g_ = 90, 70, 50, and 30 V). (c) Two-terminal field-effect mobility of monolayer MoS_2_ as a function of temperature. (d) Arrhenius plots extracted from transfer curves of monolayer MoS_2_ FETs with vdW Au electrodes. (e) Schottky barrier height extracted from activation energy (*E*_a_) as a function of gate voltage, yielding a value of approximately 9 meV.

For the WSe_2_ diode with asymmetric metal contacts, we extracted the SBH at both the Au/WSe_2_ and Al/WSe_2_ interfaces from a representative device. As shown in Figure S4a,b, the SBH for the Au/WSe_2_ interface is approximately 0.1 eV, while that for the Al/WSe_2_ interface is about 0.65 eV. These values are consistent with the observed asymmetry in the *I–V* characteristics of the WSe_2_ diodes. Furthermore, the slope of the SBH versus metal work function, shown in Figure S4c, is approximately 0.6, indicating a significant reduction in the FLP at the interface between the transferred metals and the MOCVD-grown TMD films.

### 2.2 Contact Resistance

The transmission line method (TLM) architecture was then employed to quantitatively analyze the contact resistance (*R*_C_). This analysis was performed on three vdW Au/MoS_2_ devices with identical geometries (Figure 3a). Additionally, three control devices with evaporated Au contacts were fabricated for comparison (Figure S5). As illustrated in Figure 3b, the two-terminal resistance (*R*_2T_) of MoS_2_ devices with vdW contacts exhibits a linear dependence on channel length under a gate bias of 60 V. In contrast, the *R*_2T_ of MoS_2_ devices with evaporated contacts remains largely independent of channel length and is a few orders of magnitude higher than that of the vdW-contacted devices at the same gate bias. These results indicate that vdW electrodes yield low contact resistance, whereas the evaporated electrodes introduce significant contact resistance, thereby dominating the overall two-terminal resistance. The *R*_C_ was then extracted at different carrier densities, where the carrier density was estimated by $\frac{C_{ox}}{q}\cdot \left(V_{g}-V_{th}\right)$ , with *C*_ox_ representing the capacitance per unit area of the 300 nm-thick SiO_2_, *q* representing the elementary charge, and *V*_th_ being the threshold voltage obtained by linear extrapolation for each channel. The extracted *R*_C_ decreased from 12.1 to 0.4 kΩ·μm as the carrier density *n*_2D_ increased from 1.8 × 10^12^ cm^–2^ to 6.1 × 10^12^ cm^–2^ (Figure 3b,c). The monolayer MoS_2_ FET with transferred electrodes exhibited a remarkably low *R*_C_ of 0.4 kΩ·μm at a carrier concentration of *n*_2D_ = 6.1 × 10^12^ cm^–2^, nearly 3 orders of magnitude lower compared to MoS_2_ FET with evaporated contacts.^41^ Two additional devices also demonstrated low *R*_C_ values of around 0.45 kΩ·μm at *n*_2D_ = 6.1 × 10^12^ cm^–2^ (device B) and *n*_2D_ = 5.4 × 10^12^ cm^–2^ (device C), further confirming the reliability of this contacting strategy (Figures 3d and S6). Details of the TLM device measurements are given in the Supporting Information.

**Figure 3:**
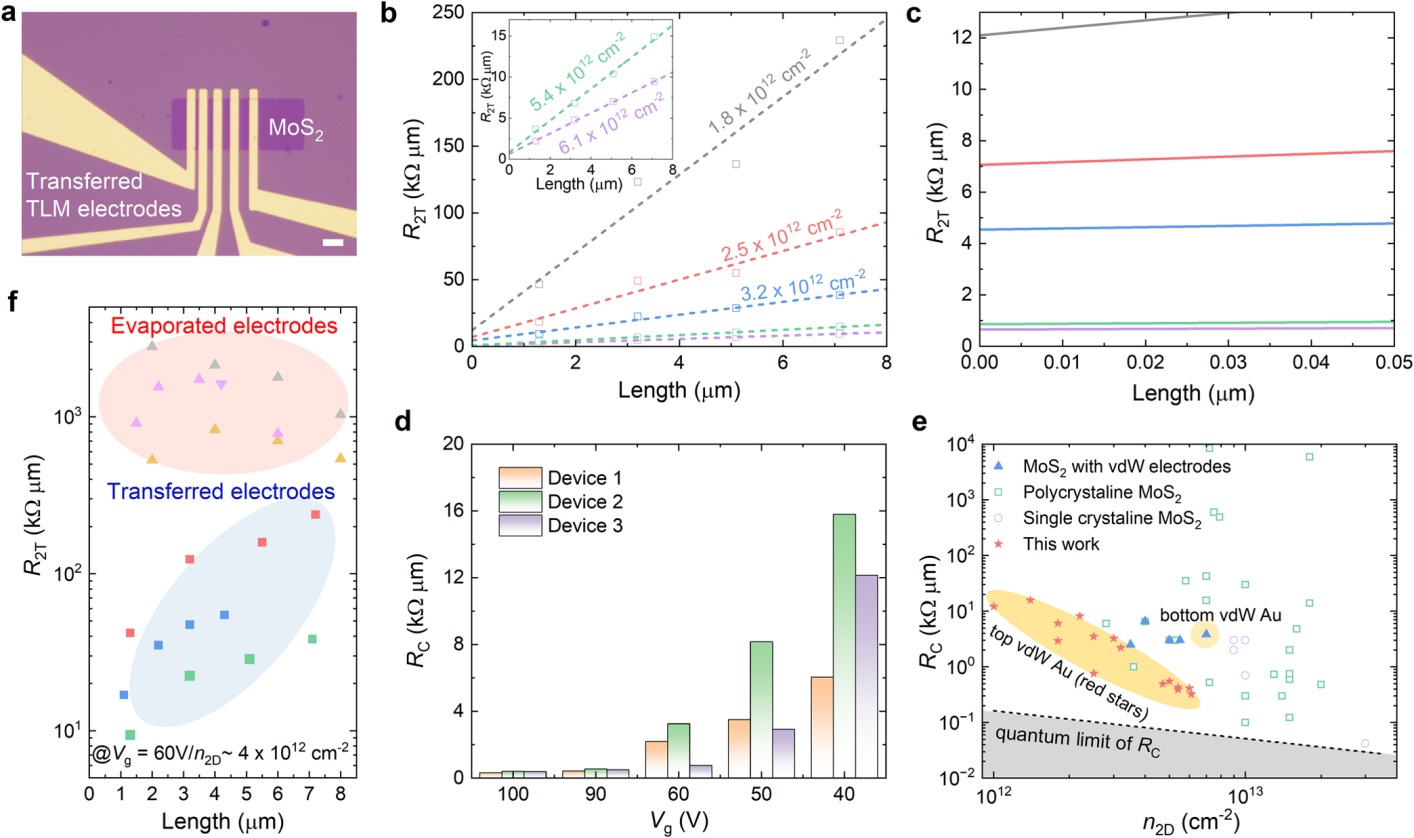
Contact resistance of MoS_2_ transmission line measurement (TLM) devices with transferred Au and evaporated Au electrodes. (a) Optical microscope image of a TLM structure device with channel lengths (*L*_c_) of 1.3, 3.2, 5.1, and 7.1 μm. Scale bar, 10 μm. (b) Two-terminal resistance of vdW-contacted MoS_2_ as a function of channel length at different carrier densities. The inset shows an enlarged area of data at carrier densities of 5.4 × 10^12^ cm^–2^ and 6.1 × 10^12^ cm^–2^. (c) Magnified data plot from (b) near *L*_c_ = 0, where the y-intercept and *x*-intercept represent 2*R*_C_, respectively. (d) Extracted contact resistance of three TLM devices with transferred electrodes at different gate voltages (*V*_g_ = 100, 90, 60, 50, and 40 V). (e) Comparison of state-of-the-art contact technologies for MoS_2_-based FETs, showing *R*_C_ as a function of carrier concentration for single crystalline and polycrystalline films, as well as other vdW electrode technologies. Our work demonstrates relatively excellent *R*_C_ for MoS_2_ with transferred electrodes. The solid black line represents the quantum limit for contact resistance. References are listed in supporting Table S2. (f) Total resistance distribution of Au–MoS_2_ contacts with transferred electrodes (blue region) and evaporated electrodes (red region) for different *L*_c_ (1–8 μm) under *V*_g_ = 60 V and *n*_2D_ ≈ 4 × 10^12^ cm^–2^, demonstrating low and consistent contact resistance in devices with transferred electrodes.

Next, we benchmarked the performance of these three TLM devices against that of previously reported MoS_2_ devices with different contacting strategies. We summarized the *R*_C_ of monolayer MoS_2_-based FETs, including single-crystalline and polycrystalline variants in Figure 3e and Table S2. Several low work-function metals or semimetals, such as Sn, Bi, In, and Sb, have been demonstrated as excellent contacting candidates for MoS_2_ to achieve relatively low *R*_C_ (even approaching the quantum limit) at high carrier concentration (∼10^13^ cm^–2^).^17,42^ Leveraging our diamond transfer method, the achieved *R*_C_ was among the best-reported values for vdW contacts, achieving within a factor of 2 from the theoretical quantum limit at a low *n*_2D_ (∼5 × 10^12^ cm^–2^). Notably, our *R*_C_ values were not only lower than those reported for polycrystalline MoS_2_-based FETs at similar or lower carrier densities^20,41,43^ but also outperformed MoS_2_ devices utilizing other bottom vdW electrodes at room temperature.^34^ To further reduce contact resistance, future improvements in material quality or the integration of high-*κ* dielectrics, such as HfO_2_ or Al_2_O_3_, could be explored.

### 2.3 Statistical Assessment of the FET Array

In commercial device manufacturing, both high yield and reproducibility are essential to achieve consistent and scalable production. To access these two key factors on the proposed technology in this work, a 10 × 10 FET array based on monolayer MoS_2_ was fabricated. Figure S2c shows the optical images of the 10 × 10 FET array fabricated on MOCVD-grown MoS_2_ with vdW Au contacts. Figure 4a displays the transfer curves of 100 MoS_2_ FETs, and all devices are functionable and exhibit the typical *n*-type characteristics, and the on-state current is around 10^–6^ A μm^–1^ at a gate voltage of 50 V with a *V*_ds_ of 1 V, which is comparable to that obtained by polycrystalline MoS_2_ films.^41^ In accordance with Figure 4b, the device array exhibited excellent uniformity, which is superior to the controlled device array with evaporated contacts (Figure S7). Figure 4b–f summarizes the device-to-device variation in terms of the on/off ratio, field-effect mobility, subthreshold swing (SS), and threshold voltage (*V*_th_). The statistical analysis shows that the average on/off ratio is 5.1 × 10^5^. In addition, all FETs showed transistor operation with an average carrier mobility of ∼30 cm^2^ V^–1^ s^–1^, which is comparable with other wafer-scale homogeneous MoS_2_ film.^44^ The SS and *V*_th_ values from 100 transistors (Figure 4d, e) exhibit narrow, centralized distributions with mean values of 2.2 V dec^–1^ and 1.9 V, respectively, manifesting the excellent reproducibility of the present transfer method. The large subthreshold swing values compared to the thermodynamic limit (∼60 mV dec^–1^) are a result of the thick (300 nm) oxide gate dielectric used here and could be improved with a thin gate dielectric. For the controlled device array, by using the traditional metal evaporation method on the same MOCVD-grown MoS_2_ film, we observed a relatively wider distribution in on/off ratio, mobility, and SS (Figure S8). The average field-effect mobility is around 7 cm^2^ V^–1^ s^–1^, smaller than that of devices fabricated by transferred electrodes, which highlights the superior performance achieved through our diamond-assisted transfer technique. In addition, the mappings of these key parameters in Figure S7 suggest the devices in the center area are more uniform than the ones along the edges. This discrepancy could be attributed to the strain formation during the transfer process.^45,46^ Our results, along with the corresponding device-to-device variation, are on par with the literature-reported large-area-grown MoS_2_ polycrystalline film (Table S3).

**Figure 4:**
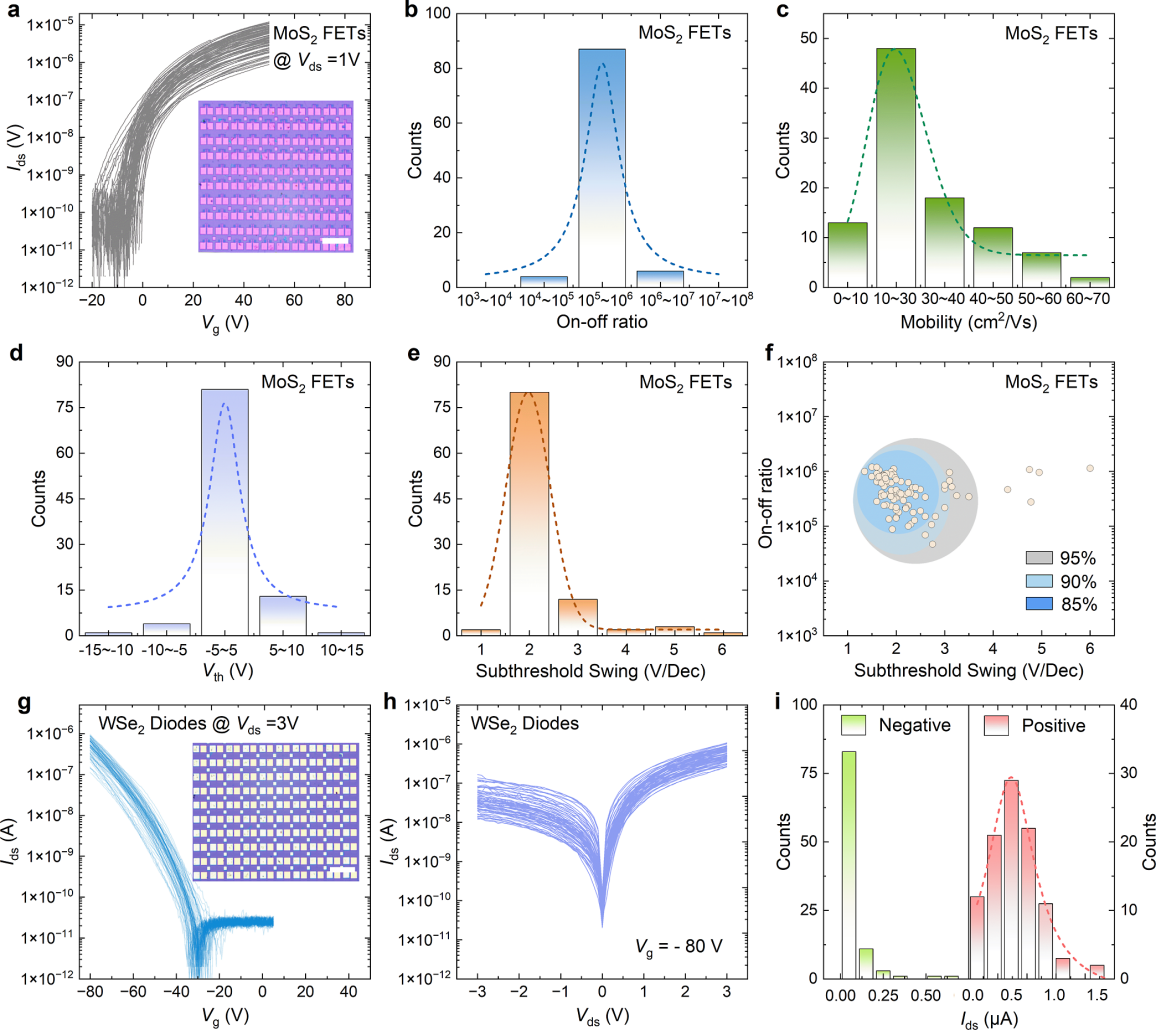
Device-to-device variation in the characteristics of the MoS_2_ transistor array and the WSe_2_ Schottky diode array. (a) Transfer curves of 100 MoS_2_ FETs with vdW Au contacts measured at *V*_ds_ = 1 V under dark conditions. Inset is the optical image of the MoS_2_ FET array. Scale bar, 400 μm. (b,e) Histogram and Lorentz distribution of 100 transistors showing (b) on/off ratio, (c) electron field-effect mobility, (d) threshold voltage, and (e) subthreshold swing (SS). (f) Statistics distribution of SS values. (g) Transfer curves of 80 WSe_2_ Schottky diodes at *V*_ds_ = 3 V. The inset shows the optical image of the WSe_2_ Schottky diode array. Scale bar, 400 μm. (h) Output curves of these diodes. (i) Histogram distribution of source-to-drain current at negative and positive contacts under a 3 V gate bias.

In addition, to demonstrate the adaptability of this technique for Schottky diode applications, an array of back-gated Schottky diodes based on monolayer WSe_2_ with asymmetric contacts was fabricated (Figure S2b). The devices were constructed by using a one-step asymmetric contact transfer strategy employing Al (low work function) and Au electrodes (high work function). This approach effectively minimizes potential contamination at the electrode-material interfaces compared with multistep transfer processes. The transfer curves presented in Figure 4g reveal that all devices consistently exhibit *p*-type behavior and high uniformity, with the gate voltage response shifting toward the negative bias region. Mapping of the *I*_on_ and *I*_off_ from the diodes is shown in Figure S9, indicating relatively uniform current distribution. Furthermore, the output characteristics shown in Figure 4h display clear rectification behavior. Specifically, at positive bias, the forward current (*I*_pos_) demonstrates a narrow distribution within the range of 0.5–1.5 × 10^–6^ A, while the reverse current (*I*_neg_) remains confined to the range of 0.001–0.025 × 10^–6^ A (Figure 4i). These results highlight the potential of the diamond transfer technique for the scalable fabrication of Schottky barrier diode arrays with high device uniformity. It should be noted that both the MoS_2_ FET array and WSe_2_ array only experienced a slight degradation after one year (Figure S10), demonstrating the excellent long-term stability and durability.

### 2.4 Photodetection Performance

We also investigated the pixel-to-pixel photoresponse variation of the MoS_2_ FET array under light illumination. Figure 5a shows the transfer curves of 100 phototransistors before and after exposure to white light with a power intensity of 30 W m^–2^ at *V*_g_ of −15 V and *V*_ds_ of 1 V. The corresponding histograms in Figure 5b illustrate the distribution of dark current and photocurrent upon light illumination. The mean photocurrent and responsivity were found to be 3.1 × 10^–8^ A and 1.6 A W^1–^, respectively (Figure 5c). To leverage the photoelectric detection capability of the transferred contact device, we conducted visible-blind imaging. A 10 × 10 array was programmed to display the pattern information “MUST” with the light source selectively controlled (Figure 5d). When the light was turned on and off, the devices responded by outputting the corresponding current signals, which were collected and processed. The resulting output pattern with spatial uniformity, thanks to the uniform devices shown in Figure 5e, clearly demonstrates the potential of vdW-contact-based phototransistors for imaging applications.

**Figure 5:**
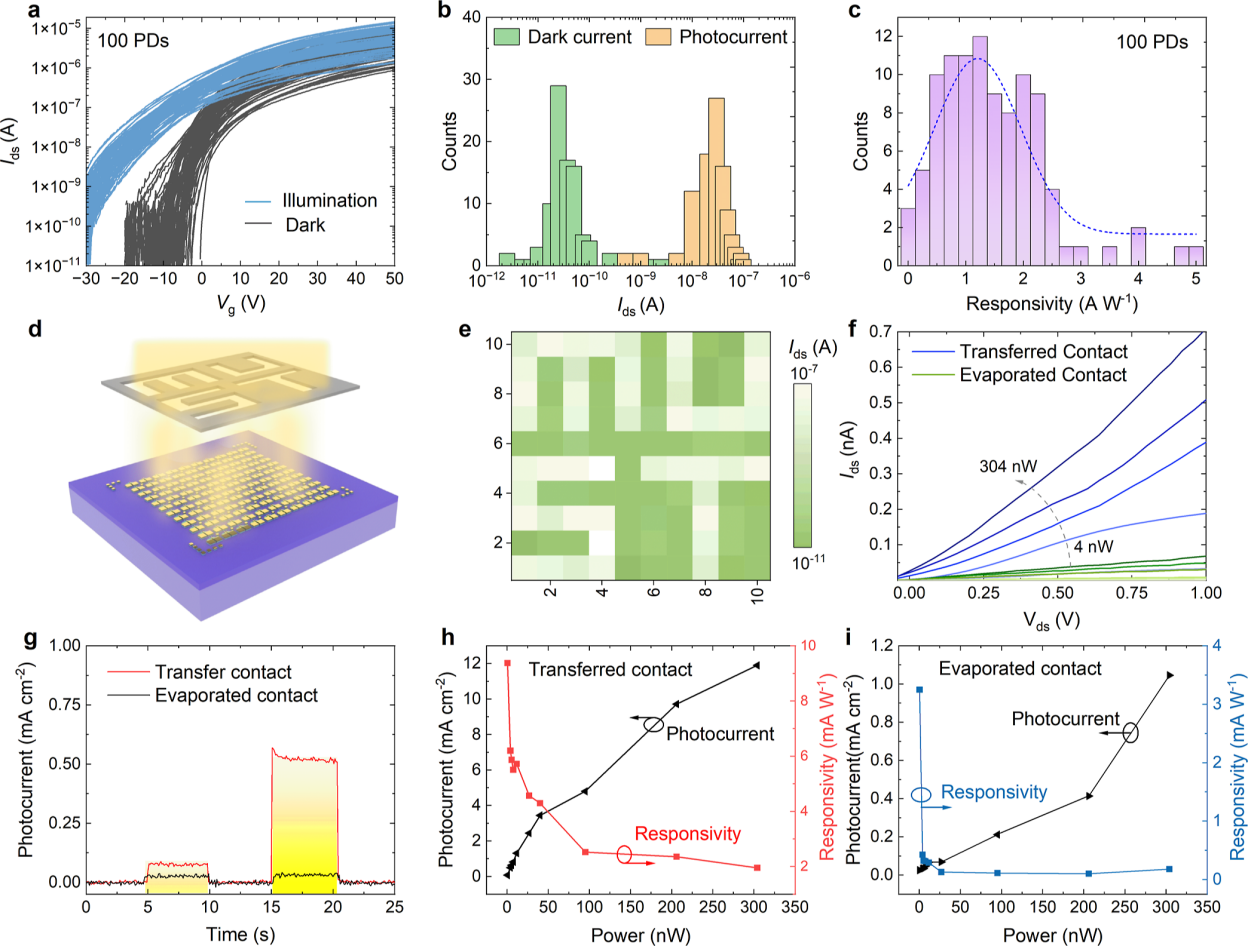
Phototransistor performances. (a) Transfer characteristics of 100 MoS_2_ phototransistors before and after exposure to white light (30 W m^–2^) at *V*_g_ = −15 V. (b) Histogram of dark current (green) and photocurrent (yellow). (c) Histogram and Gaussian distribution of photoresponsivity. (d) Schematic of the measurement set up for the photodetection array. (e) Imaging pattern of the vdW- electrode MoS_2_ device with the input image displaying the characters “MUST”. (f) Current–voltage curves of WSe_2_ phototransistors under 532 nm laser illumination (0.4 and 4 nW), comparing transferred and evaporated contacts. (g) Photocurrent response under on/off laser illumination. (h,i) Photocurrent and photoresponsivity of WSe_2_ phototransistors with transferred and evaporated contacts.

To better compare the optoelectronic properties of evaporated-contact and vdW-contact devices, we performed standard single-wavelength-laser-based photoresponse measurements. Figure 5f shows the current–voltage characteristics of the WSe_2_-based phototransistor in the dark and under 532 nm laser illumination. Both device types exhibited an increase in current with increasing illumination intensity, confirming a power-density-dependent photocurrent response. However, compared to the evaporated-contact device, the transferred-contact device significantly suppressed the dark current while enhancing the photocurrent. To further investigate the photoresponse behavior, time-dependent photoresponse measurements were performed under a fixed 1 V bias, showing two representative cycles (Figure 5g). The extracted photocurrent and calculated responsivity (Figure 5g,h) clearly indicate that the transferred-contact device exhibits a higher photocurrent and superior responsivity compared to the evaporated-contact device.

## 3 Conclusions

In summary, we demonstrated that the hydrogenated diamond-assisted electrode transfer technology can be effectively adopted for the scalable and reliable fabrication of large-scale 2D semiconductor electronics and optoelectronics. We have shown that this strategy could be utilized to fabricate 100% yield high-performance poly-MoS_2_ FET arrays with high consistency. The average field-effect mobility is ∼30 cm^2^ V^–1^ s^–1^, with the highest device showing 79 cm^2^ V^–1^ s^–1^. The lowest contact resistance is 400 Ω μm at a low carrier density of 6.1 × 10^12^ cm^–2^, which benefits from the residual free metal–semiconductor interface. Lower device-to-device variation was achieved compared with the devices made with evaporated contacts, which offered a pathway for high-performance monolithic integrated circuits and imaging applications. We also realized the transfer of asymmetric contacts in one-step transfer and demonstrated a highly uniform Schottky diode array. In addition, high photocurrent and responsivity were demonstrated in these vdW contact arrays, offering the potential for image detection. Our work provides a reliable pathway to develop wafer-scale vdW fabrication for future electronics and optoelectronics.

## 4 Experimental Section

### 4.1 Synthesis and Characterization of the Monolayer TMD Film

MoS_2_ monolayer was deposited on a 2-in. sapphire substrate by metal–organic chemical vapor deposition (MOCVD) in a home built MOCVD system (see Figure S1a).^47^ The reactor is a 250 mm diameter hot-wall tube furnace, with molybdenum hexacarbonyl (Mo­(CO)_6_) and hydrogen sulfide (H_2_S) as molybdenum and sulfur precursors, and the growth temperature of MoS_2_ polycrystalline film was set at 900 °C, using Ar as the carrier gas. The preparation of WSe_2_ thin films was similar to the growth of MoS_2_ films using the MOCVD method. The W­(CO)_6_ and H_2_Se were used as precursors, and the growth temperature is 800 °C.

The MOCVD MoS_2_ film was transferred to the SiO_2_ (285 nm)/Si substrate from sapphire by a wet chemical transfer process. First, the MoS_2_ on the sapphire substrate was spin-coated with PMMA (495 A4) at 2000 rpm spin speed for 60 s and heated at 180 °C for 5 min. The corners of the spin-coated film were scratched using a blade and immersed inside an HF solution for 15 min. Capillary action caused the HF to be preferentially drawn into the substrate/interface and separated the PMMA/MoS_2_ from the sapphire substrate due to the hydrophilic nature of sapphire and the hydrophobic nature of MoS_2_ and PMMA. Then, the PMMA/MoS_2_ film was picked up from the HF solution and rinsed in a water bath for 5 min and repeated two times to remove residues. The SiO_2_/Si substrates were sequentially placed in acetone, absolute ethanol, and deionized water for ultrasonic cleaning for 10, 10, and 5 min, respectively, and dried with a nitrogen gas gun. The PMMA/MoS_2_ film was then picked up by the SiO_2_ substrate and followed by vacuum drying for 1 h. After that, the PMMA/MoS_2_ on SiO_2_/Si was heated on a hot plate at 180 °C for 1 min. Acetone was used to remove PMMA and leave a clean surface of monolayer MoS_2_ on the SiO_2_/Si substrate.

Transmission electron microscopy (TEM) and selected area diffraction images were acquired by using an FEI Tecnai F20 system operated at an accelerating voltage of 200 kV. Figure S1h displays a continuous monolayer film and one set of hexagonal symmetrical patterns, indicating the highly crystalline nature of the MoS_2_ films. False-color processing was applied to the dark-field TEM images, as shown in Figure S1i,j, showing that the film grain domain size is 1–3 μm. Figure S1c,d depicts the optical image of the monolayer MoS_2_ film as grown on sapphire and transferred on SiO_2_, presenting a continuous film over a large area. Raman spectra of MoS_2_ film (Figure S1i) confirm the growth on sapphire substrate and transfer on the SiO_2_/Si substrate have in-plane (E^1^_2g_) and out-of-plane (A_1g_) modes at 386 and 404 cm^–1^, respectively. Mapping of the two peaks on the transferred MoS_2_ film over a 25 mm^2^ area and peak separations of 18 cm^–1^ for as-grown and transferred MoS_2_ films reveals that the transfer process maintains the quality of this monolayer MoS_2_ film (Figure S1j,k). The variation of peak separation falls within a narrow range (18.03 ± 0.128 cm^–1^) indicating that the film is uniform across the measured area.

### 4.2 Device Fabrication

The diamond substrates were polished by a scaif wheel (Technical Diamond Polishing) to reduce surface roughness and hydrogen terminated before metal deposition.^32^ The hydrogen termination was performed in a *Seki 6500* diamond deposition reactor with exposure to a hydrogen plasma of 85 Torr, 4500 W at 800 °C with an H_2_ flow rate of 450 sccm. Electrode arrays were fabricated on hydrogenated diamond substrates using standard photolithography, e-beam evaporation, and a lift-off process. Symmetric Au contacts were chosen for MoS_2_ FETs, while asymmetric contacts comprising a high-work-function metal (Au) and a low-work-function metal (Al) were used for WSe_2_ Schottky diodes. All electrodes were 30 nm thick and were deposited at a low deposition rate of ∼0.3 Å·s^–1^ (∼10^–6^ mbar). After the lift-off process, the patterned metals on hydrogenated diamonds were picked up by using polycarbonate (PC) stamps on a glass slide. The PC stamp and the diamond with metal contacts were both heated from room temperature to 150 °C with a heating rate of ∼15 °C min^–1^. It should be noted that when the temperature increased, the PC film became adhesive as it transitioned from a hard state to a more rubbery state. The PC film conformed to all the geometry of the electrodes on the diamond substrate when the temperature reached 150 °C and then cooled down to room temperature to delaminate electrodes from the diamond surface. All the prepatterned metal contacts can be fully detached from the diamond, leaving the diamond surface clean with no visible metal flakes or polymer residue. The distance between metal electrodes can be reduced down to 500 nm, as shown in Figure S2d. An optical microscope, along with a micromanipulator, was used to align and transfer the metal electrode array to the desired region of the TMD film. Once the temperature reached 180 °C, the PC stamp with metal contacts was released entirely, leaving the PC/electrode array on the TMD film. The PC film was then removed by soaking in CHCl_3_ at 60 °C. Finally, a photolithography process was applied to define the active channels with the help of square block alignment markers to ensure precise pattern projection. Reactive ion etching with oxygen gas was then used to remove the unprotected TMDs. The remaining photoresist was removed using dimethyl sulfoxide and deionized water. Similarly, electrodes for TLM devices were fabricated by using the above process. Different channel lengths (*L*), varying from 1 to 8 μm between neighboring electrodes, were designed to measure the Au/MoS_2_ contact resistance. The rectangular monolayer MoS_2_ regions were defined and patterned by direct UV lithography and the previously described RIE process.

### 4.3 Electrical Transport Characterizations

The transport behaviours of the MoS_2_ transistor array with vdW electrodes and the TLM device were measured in a model TTPX Cryogenic Probe Station in a high-vacuum environment with a base pressure of 10^–6^ mbar. Temperature-dependent transport measurements were performed in the range of 300–77 K using a TeslatronPT Oxford Cryostat. Two Keithley 2400 source meters were employed to apply *V*_ds_ and *V*_gs_, while monitoring *I*_ds_ and gate leakage current.

### 4.4 Optoelectronic Measurements

The measurements were performed using a confocal microscope system (WITec alpha 300R) with a 50× objective lens (NA = 0.9) under ambient conditions. A 532 nm laser was coupled through a fiber bench with an optical chopper to adjust the power intensity with a spot size of 1 μm in diameter. The samples were illuminated from the top side on a piezo-crystal-controlled scanning stage. A source meter was employed to apply the source-drain voltages and collect the photocurrent data.

## Supplementary Material



## Data Availability

All data supporting the findings of this study are available from the corresponding author upon reasonable request.

## Notes

Comparison of different metal transfer technologies, schematic illustration of MOCVD-grown TMDs and corresponding thin-film characterizations using TEM and Raman spectroscopy, fabrication procedures for device arrays along with representative optical images, temperature-dependent transfer curves of a MoS_2_ FET, SBH extraction for a WSe_2_ diode, output characteristics (*I–V* curves) of TLM devices under different gate voltage, contact resistance measurements for TLM devices B and C, benchmarking of contact resistance in MoS_2_ based FETs using state-of-the-art contact technology, spatial mapping of the key electrical parameters across the MoS_2_ FET array, electrical characterization of the controlled MoS_2_ FET array, spatial mapping of the on- and off-current in WSe_2_ Schottky diode array, comparative analysis of carrier mobilities of MoS_2_ fabricated using different methods, and long-term stability assessments of both the MoS_2_ FET and WSe_2_ diode arrays (PDF)
