# Supplementary material for: One-Step Transfer of Symmetric and Asymmetric Contacts for Large-Scale 2D Electronics and Optoelectronics
Source: ACS Nano. 2025 Jul 23;19(30):27919–29. doi: 10.1021/acsnano.5c09815 (PMC12333430; doi:10.1021/acsnano.5c09815)
Supplement: Supplementary file 1 [file nn5c09815_si_001.pdf]

One-step transfer of symmetric and asymmetric contacts for large-scale 2D electronics and optoelectronics

Jingying Liu[1,2,†], Kaijian Xing[1,2,3†*], Lintao Li[4,†], Weiyao Zhao[5], Alastair Stacey[6,7], Islay Robertson[6], David A. Broadway[6], Jean-Philippe Tetienne[6], Dong-Chen Qi[8], Michael S. Fuhrer[3], Yufeng Hao[4*], Qingdong Ou[1,2*]

[1] Macau University of Science and Technology Zhuhai MUST Science and Technology Research Institute, Zhuhai, 519031, China

[2] Macao Institute of Materials Science and Engineering (MIMSE), Faculty of Innovation Engineering, Macau University of Science and Technology, Taipa, Macao, 999078, China

[3] School of Physics and Astronomy, Monash University, Clayton, Victoria 3800, Australia

[4] National Laboratory of Solid State Microstructures, College of Engineering and Applied Sciences, Jiangsu Key Laboratory of Artificial Functional Materials and Collaborative Innovation Center of Advanced Microstructures, Nanjing University, Nanjing 210023, China

[5] Department of Materials Science & Engineering, Monash University, Clayton, Victoria 3800, Australia

[6] School of Science, RMIT University, Melbourne, Victoria 3000, Australia

[7] Princeton Plasma Physics Laboratory, 100 Stellarator Road, Princeton, New Jersey 08540, USA

[8] Centre for Materials Science, Queensland University of Technology, Brisbane, Queensland 4001, Australia

†J.L., K.X., and L.L. contributed equally to this work.

*Correspondence to: kaijian.xing@monash.edu; haoyufeng@nju.edu.cn; qdou@must.edu.mo

Supporting Table S1. Comparison of different metal transfer technologies.

Method	*Wafer scale*	*Without sacrificed layer*	*Reusability*	*Asymmetric contact*	*Photolithography compatibility*	*Ref.*
Hexamethyldisilazane (HMDS)-assisted	No	No	No	No	Yes	1
hBN	No	No	No	No	Yes	2
HMDS	No	No	Not mentioned	No	Yes	3
HMDS	No	No	Not mentioned	No	Yes	4
PVA	No	No	Not mentioned	No	Yes	5
ZnO nanobelt	No	No	No	No	No	6
Graphene	Yes	No	No	No	Yes	7
Polypropylene carbonate	Yes	No	No	No	No	8
PMMA	Yes	No	No	No	Yes	9
Fluorphlogopite	Yes	Yes	Not mentioned	No	Yes	10
Hydrogenated Diamond	Yes	Yes	Yes	Yes	Yes	This work

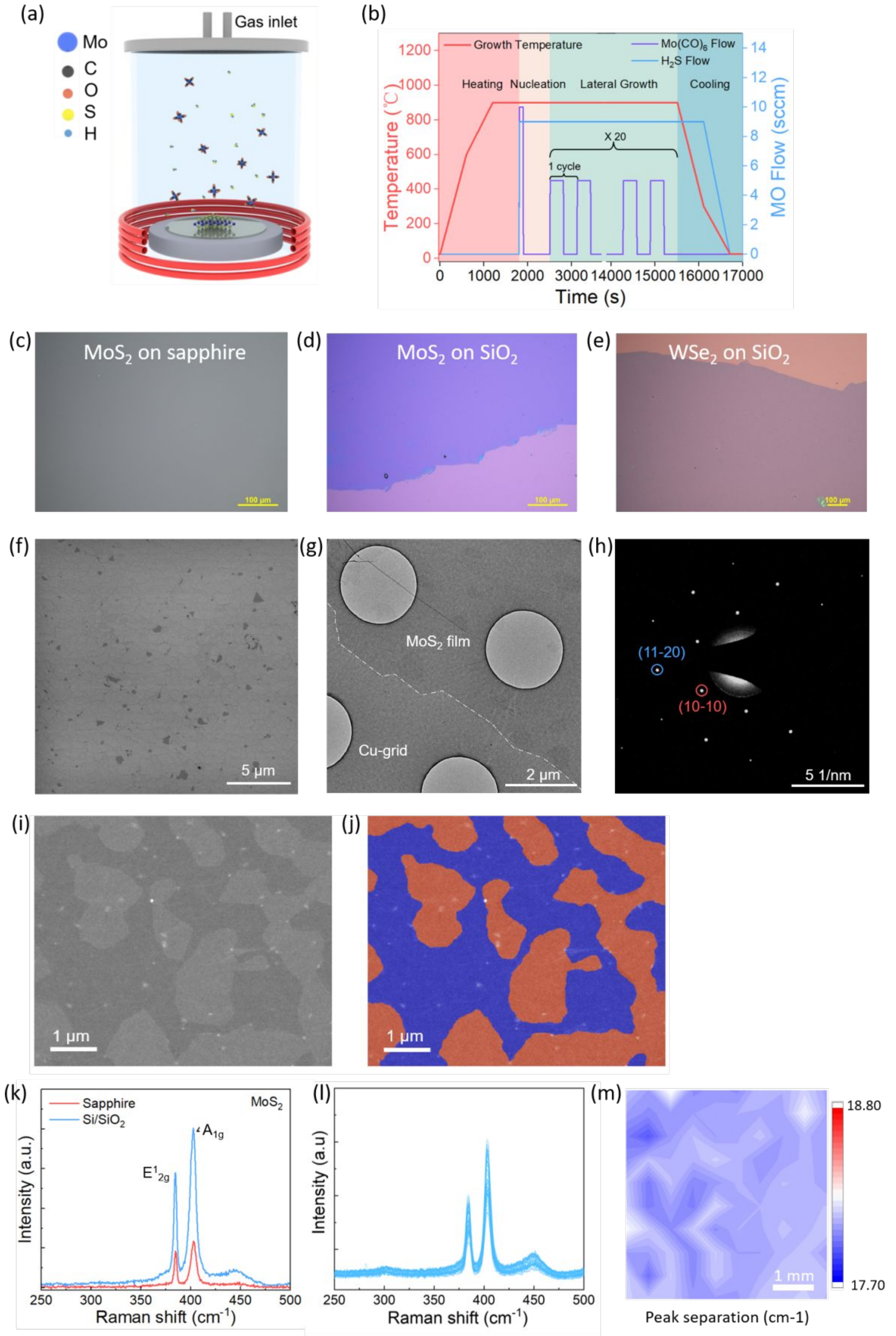

Figure S1. (a) Schematics of MOCVD growth setup for the synthesis of monolayer MoS_2 on sapphire substrate. (b) Temperature profile and MO gas flow in the MOCVD growth. (c-e) Optical image of MoS_2 on sapphire, MoS_2 on SiO_2, and WSe_2 on SiO_2. (f-h) TEM images and selected-area diffraction TEM pattern of monolayer MoS_2 film. (i) The dark-field TEM image of MoS_2 film by MOCVD, (j) The corresponding false-color image. (k) Raman spectrum of as-grown monolayer MoS_2 on sapphire on SiO_2/Si substrate after wet transfer. (l) Raman spectra measured over 5 mm × 5 mm area. (m) Corresponding spatial colour mapping of peak separation between the two Raman active modes, E^1_{2g} and A_{1g} from (l).

The dark-field TEM image was employed to characterize the domain size of MOCVD-grown MoS_2 thin films (**Figure S1i**). Domain size quantification relied on imaging contrast variations in dark-field TEM arising from differing domain orientations. For enhanced visual clarity, false-color processing was applied to the dark-field TEM images, as shown in **Figure S1j**. The resulting domain size of the grown film ranges from 1–3 μm

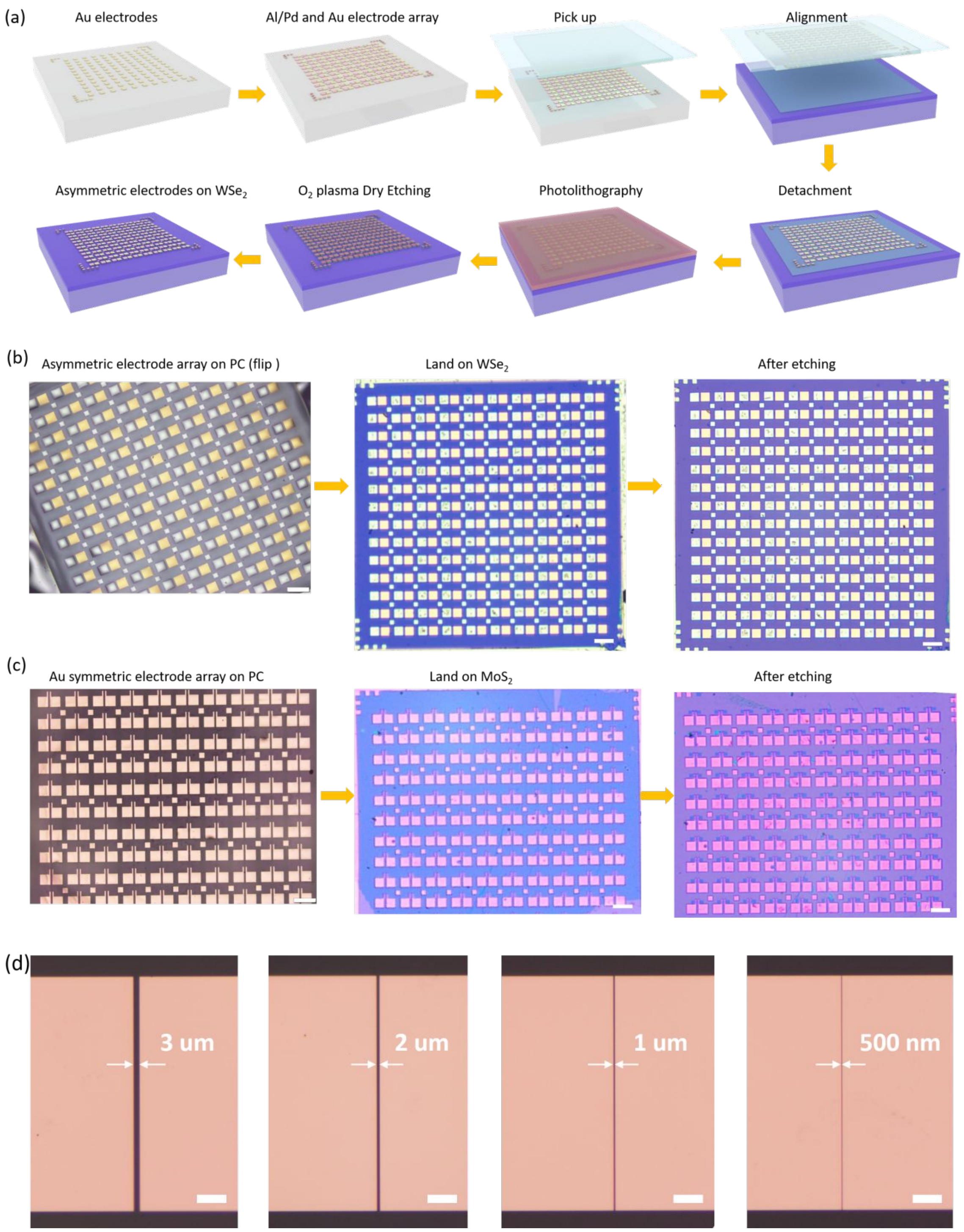

Figure S2. (a) Schematic of fabrication process for the asymetric contact array of Au and Al/Pd on MOCVD-grown WSe_2 monolayer. (b) Optical image of asymmetric contact device array picked up by polycarbonate (PC) stamp and on WSe_2 thin film before and after etching. (c) Optical image of the Au electrodes array picked up by PC stamp on MoS_2 thin film before and after etching. Scale bar, 200 μm. (d) Optical images of metal contacts with different channel length (L=3, 2, 1, and 0.5 μm) picked up by PC stamp.

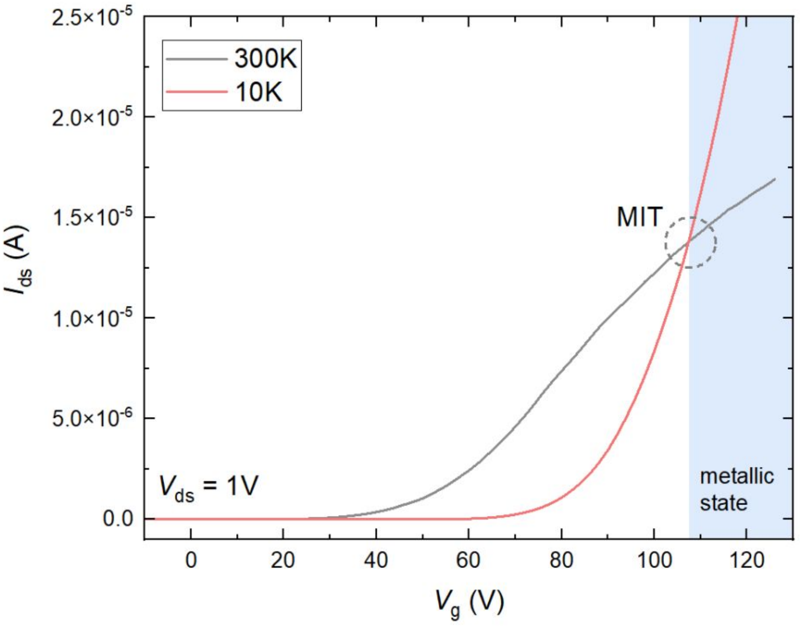

Figure S3. Temperature-dependent I_{ds}-V_{gs} transfer curves of MoS_2 FET showing metal insulating transition point at 107V gate bias. $V_{ds} = 1V$.

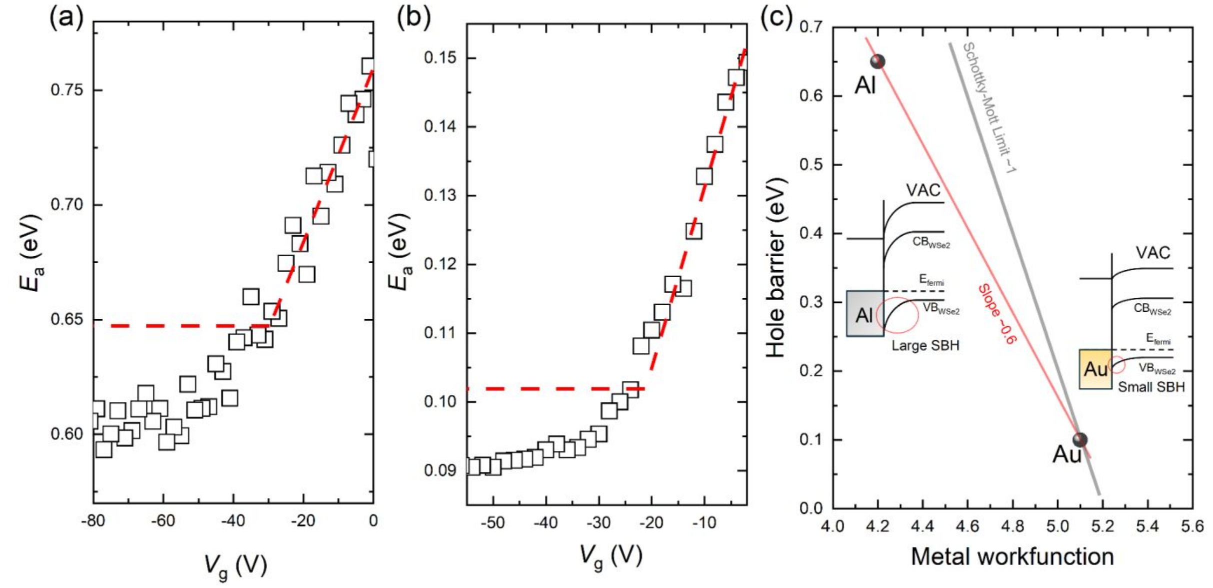

Figure S4. (a) and (b) represent SBH extracted from activation energy (E_a) as a function of gate voltage, (c) Extracted SBH for different transferred metals on MOCVD-grown WSe_2, plotted as a function of the metal work function. The solid grey line represents the Schottky-Mott limit, and the solid red line represents the linear fit for the transferred metals (slope ~ 0.6).

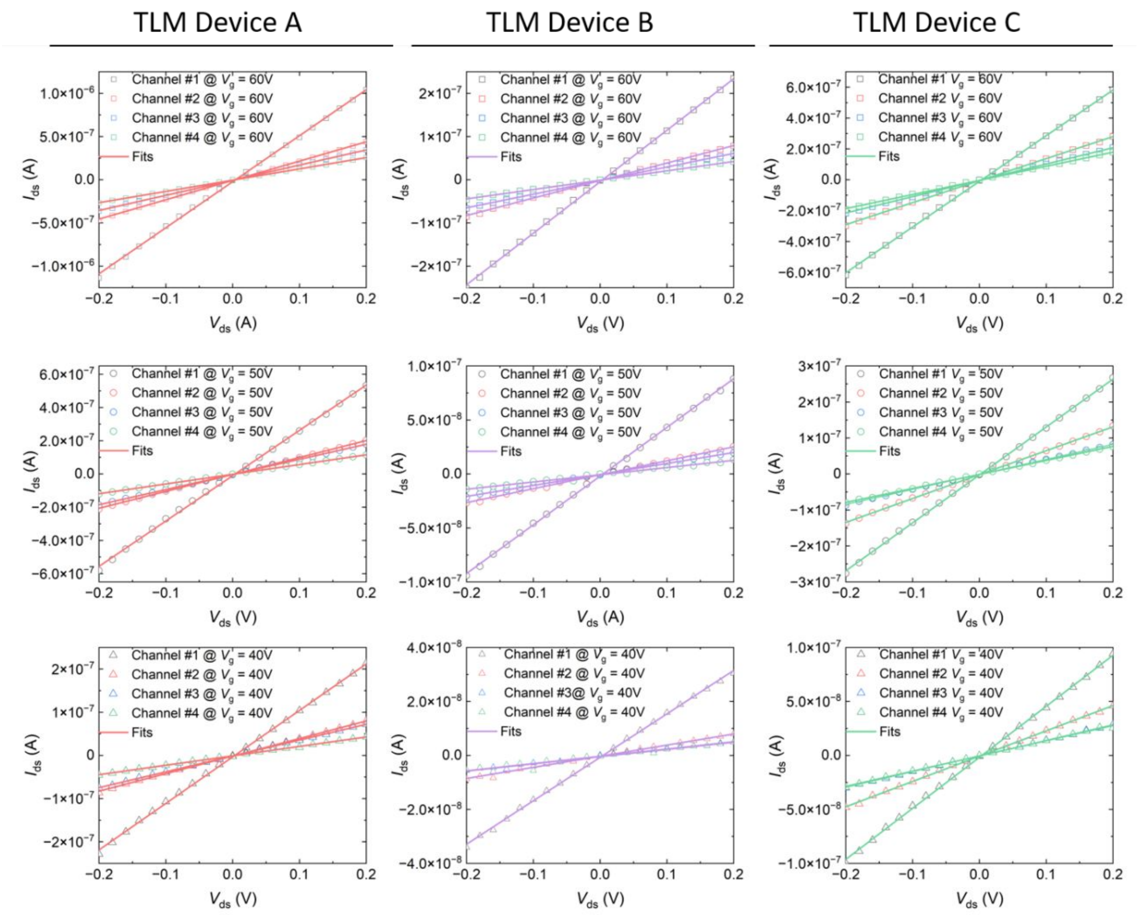

Figure S5. Output curve of the three TLM device A, B and C with transferred electrodes under different gate voltage (V_{ds} from = 0.2 to 0.2 V).

Figure S5 show the output curve curves of three TLM device with transferred Au contact for different channel lengths at back gate of 60V, 50V and 40V. The devices show a linear source-drain current versus source drain voltage output curve, suggesting the formation of a good ohmic contact with MoS_2.

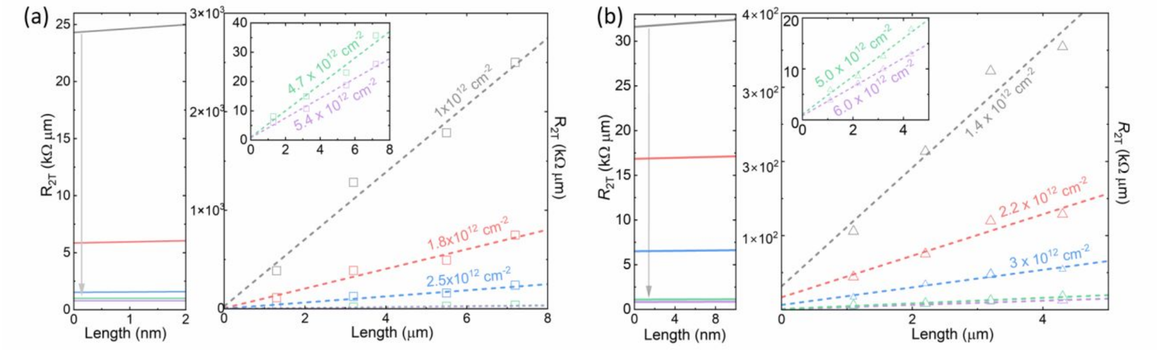

Figure S6. Rc extraction plot from device TLM device B and C with transferred electrodes. (a) TLM device B shows Rc of 12.2 kΩ·µm, 2.8 kΩ·µm, 800 Ω·µm, 500 Ω·µm, 450 Ω·µm at n_{2D}=1 × 10^{12} cm^{-2}, 1.8 × 10^{12} cm^{-2}, 2.5 × 10^{12} cm^{-2}, 4.7 × 10^{12} cm^{-2}, 5.4 × 10^{12} cm^{-2}. (a) TLM device C shows Rc of 15.7 kΩ·µm, 8.2 kΩ·µm,3.2 kΩ·µm, 550 Ω·µm and 450 Ω·µm, at n_{2D}=1.4 × 10^{12} cm^{-2}, 2.2 × 10^{12} cm^{-2}, 3 × 10^{12} cm^{-2}, 5 × 10^{12} cm^{-2}, 6 × 10^{12} cm^{-2} (From top to bottom).

By means of the TLM, the width-normalized contact resistance for device B and C is calculated to be 450 Ω·µm (n_{2D}=5.4 × 10^{12} cm^{-2}) and 450 Ω·µm (n_{2D}=6 × 10^{12} cm^{-2}).

Supplementary Table S2. State-of-the-art contact technology for contact resistance of MoS_2 based FETs.

Growth method/thickness	***Contact metals***	***Gate***	***Rc (kΩ/μm)***	***Carrier density /Vds***	***Date***	***Ref.***
Epitaxial CVD 1L on sapphire	2/30 nm Ti/Au (EBE)	EBL/global Si back gate	490 (Top-contact) V_{gs}=70V	n=7.9 x10^{12} 1V	2017	11
MOCVD 2L on SiO_2	Cr/Au, 3/30 nm (EBE)	Photo/high-k top gate	59 (Top-contact) V_{gs} = 150 V	n ≈ 1.8 × 10^{13} 1V	2018	12
CVD 1L on SiO_2	10nm In 100nm Au EBE	EBL/global Si back gate	3±0.3	n = 5.0 × 10^{12} 0.1V	2019	13
MOCVD 1L on sapphire	40 nm Ni/30 nm Au EBE	EBL/Global high-k back gate	3 (Top-contact)	n = 2.7×10^{12} 1V	2021	14
MOCVD 1L on SiO_2	20nm Bi semimetal EBE	300-nm-thick SiO_2 and100-nm-thick SiN_x	0.123	n = 1.5 × 10^{13} cm^{-2} 1V	2021	15
CVD 1L on SiO_2	20 nm Bi EBE	EBL/20nm hBN dieletric Bi Top gate	0.078 (V_{gs}-12V)	n ≈ 1.1 × 10^{13} at 15 K 0.5V	2023	16
MOCVD 1L on SiO_2	Ti/Au EBE	Photo/high k Top gate 20nm Al_2O_3 10nm HfO_2	3.8 (bottom-contact) 42.6 (Top-contact)	n = 4.3x10^{11} 1V	2024	17
Scotch-tape exfoliated 1-15 layer MoS_2	Au	EBL/global Si back gate	0.74	n = 10^{13} 1V	2016	18

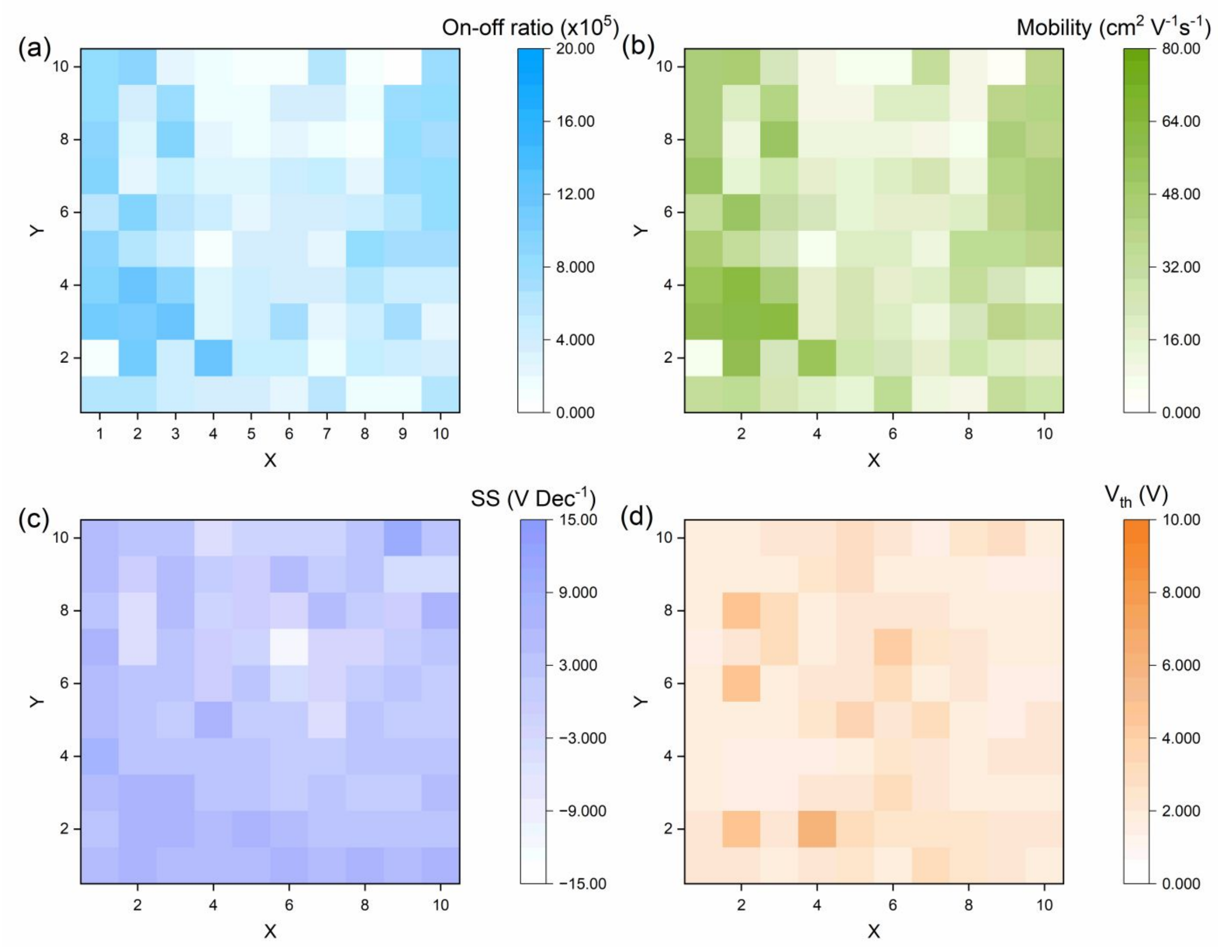

Figure S7. Mapping of the (a) on-off ratio, (b) mobility, (c) Subthreshold slopes (SS) and (d) Threshold voltage (V_{th}) from the 100 devices with transferred Au symmetric contact.

The electronic properties of 10 x 10 devices are investigated, which are derived from the transfer curves shown in Figure 4a in the main text. The field effect carrier mobility can be determined by $\mu = \frac{L}{WC_{\text{ox}}\text{V}_{\text{ds}}} \cdot \frac{dI_{\text{ds}}}{dV_{bg}}$ from the transfer curve.

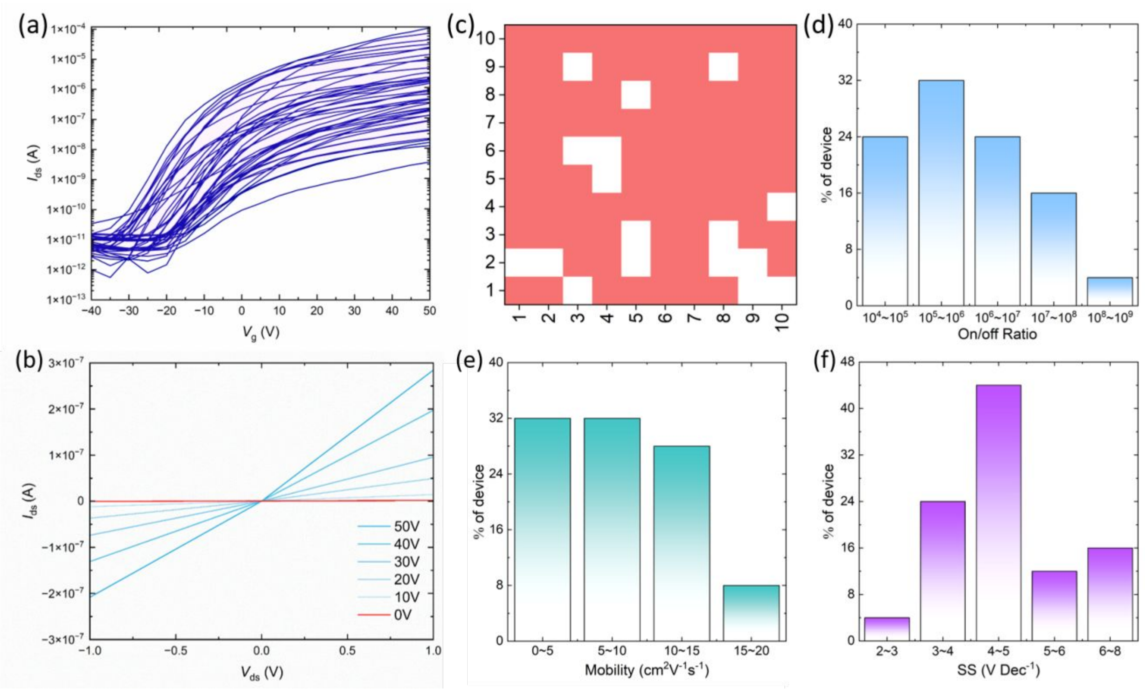

Figure S8. Device to device variation in the characteristic of MoS_2 transistor arrays made by evaporated Au electrodes. (a) Transfer characteristics, source to drain current I_{ds} as a function of the back-gate voltage V_g at a source-to-drain voltage V_{ds} of 1V and measured in the dark. (b) Output characteristics of the devices, drain current as a function of drain voltage with the back gate voltage varying from 0 to 50V in a step of 10V. (c) Die map showing the device yield. Red square indicates the device can be turned on while white means could not be turned on. (d-e) The statistic distribution of the (d) on/off current ratio, (e) Electron field-effect mobility value, and (f) SS from devices made by evaporated electrodes.

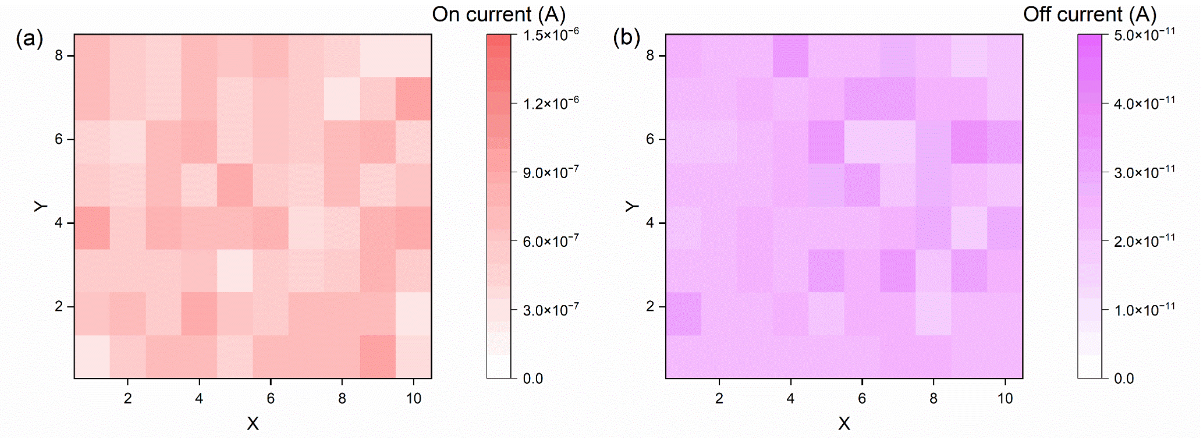

Figure S9. Mapping of the (a) on current and (b) off current from 80 schottky diode array with transferred asymmetric contacts.

Supporting Table S3. Comparison of motilities of MoS_2 fabricated by different methods.

Growth method	***Thickness/substrate***	***Mobility ($cm^2 V^{-1} s^{-1}$)***	***Contact metal***	***Fabrication method***	***Ref.***
Exfoliated	1L	50	10/40 nm Ti/Au	e-beam evaporation (EBE)	19
	1L	50	5/10 nm In/Au	EBE	20
	1L	260	Ag/Pt	Transferred electrodes	1
	1L	193 (130K)	Gr/Ag	Transferred electrodes	21
CVD	1L on SiO_2	167 ± 20	10/100nm In/Au	EBE	13
	1L on sapphire	75	20/30 nm Sb/Au	EBE	22
	1L on SiO_2	42	50 nm Au	EBE	23
	1L on SiO_2	5.9	10/70 nm Ag/Au	Transferred electrodes	5
MOCVD	1L on SiO_2	0.47	5/50 nm Ti/Au	EBE	24
	2L on SiO_2	18.1	3/30 nm Cr/Au	EBE	12
	1L on polyimide	10	Not mentioned		25
	1L on SiO_2	8	10/25-nm Ti/Au	EBE	26
	1L on sapphire	3.6 - 23.9	40/30 nm Ni/Au	EBE	14
	1L on SiO_2	120 at 77 K and 55 at RT	20nm Bi semimetal	EBE	15
	1L on sapphire	23.6	Ni/Au	EBE	27
	2L on SiO_2	10.42	3/30 nm Cr/Au	EBE	28
	1L on SiO_2	21	30 nm Ti/Au Bottom contact	EBE	17
	1L on sapphire	30 [*]	30 nm Au	Transferred electrodes	This work
	[*] Average mobility of 30 $cm^2 V^{-1} s^{-1}$ over 100 devices at RT, with 79 $cm^2 V^{-1} s^{-1}$ (highest) and 104 $cm^2 V^{-1} s^{-1}$ at 77 K				

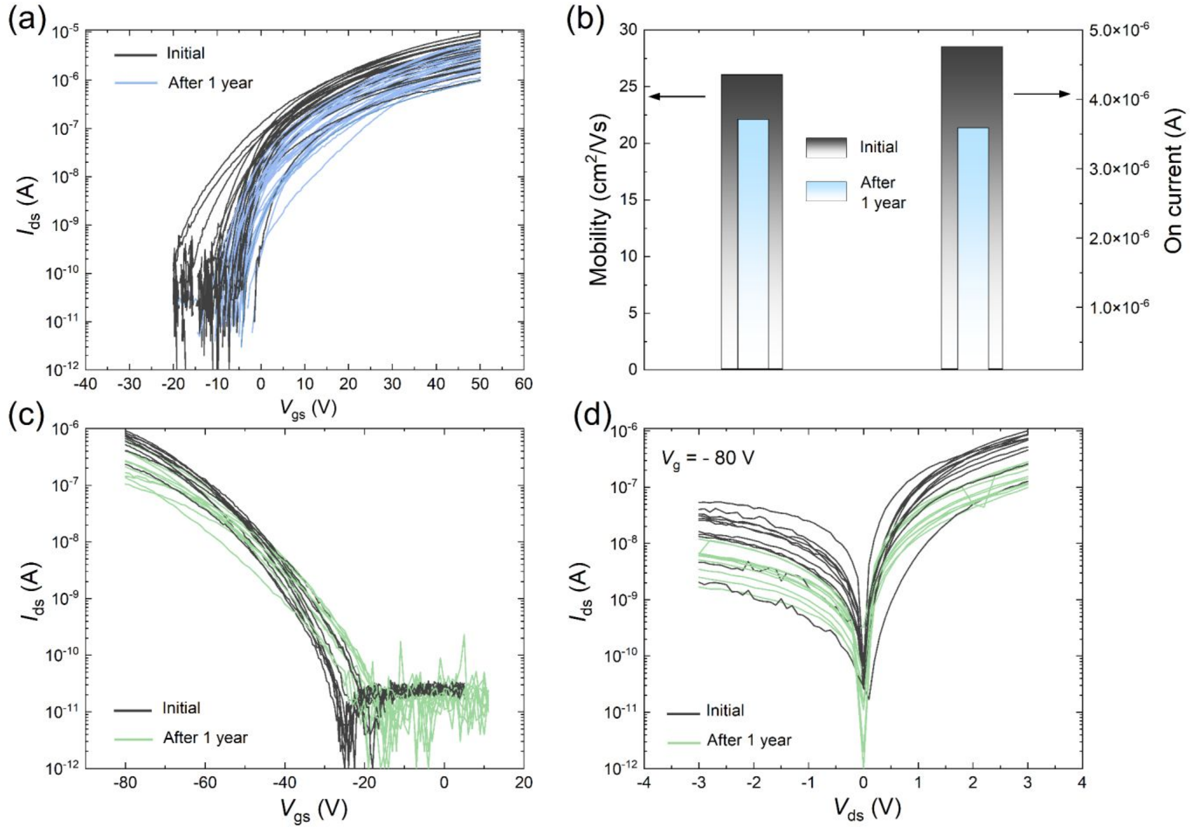

Figure S10. Long-term stability characterization. (a) Transfer curves for 30 randomly selected MoS_2 FETs. Black lines represent the initial performance, and the blue lines represent the results remeasured after one year. (b) The average mobility and on current of the selected devices. (c) Transfer curves and (d) Output curves for 10 randomly selected WSe_2 FETs. Black lines represent the initial performance, and the green lines represent the results remeasured after one year.

References

1. Liu, Y.; Guo, J.; Zhu, E.; Liao, L.; Lee, S.-J.; Ding, M.; Shakir, I.; Gambin, V.; Huang, Y.; Duan, X., Approaching the Schottky–Mott limit in van der Waals metal–semiconductor junctions. *Nature* **2018,** *557* (7707), 696-700.

2. Jung, Y.; Choi, M. S.; Nipane, A.; Borah, A.; Kim, B.; Zangiabadi, A.; Taniguchi, T.; Watanabe, K.; Yoo, W. J.; Hone, J.; Teherani, J. T., Transferred via contacts as a platform for ideal two-dimensional transistors. *Nature Electronics* **2019,** *2* (5), 187-194.

3. Liu, L.; Kong, L.; Li, Q.; He, C.; Ren, L.; Tao, Q.; Yang, X.; Lin, J.; Zhao, B.; Li, Z.; Chen, Y.; Li, W.; Song, W.; Lu, Z.; Li, G.; Li, S.; Duan, X.; Pan, A.; Liao, L.; Liu, Y., Transferred van der Waals metal electrodes for sub-1-nm MoS_2 vertical transistors. *Nature Electronics* **2021,** *4* (5), 342-347.

4. Sun, X.; Wang, D.; Wu, X.; Zhang, J.; Lin, Y.; Luo, D.; Li, F.; Zhang, H.; Chen, W.; Liu, X.; Kang, Y.; Yu, H.; Luo, Y.; Ge, B.; Sun, H., Facile formation of van der Waals metal contact with III-nitride semiconductors. *Sci Bull (Beijing)* **2024**.

5. Song, X.; Liu, Z.; Ma, Z.; Hu, Y.; Lv, X.; Li, X.; Yan, Y.; Jiang, Y.; Xia, C., PVA-assisted metal transfer for vertical WSe_2 photodiode with asymmetric van der Waals contacts. *Nanophotonics* **2023,** *12* (18), 3671-3682.

6. Hong, M.; Zhang, X.; Geng, Y.; Wang, Y.; Wei, X.; Gao, L.; Yu, H.; Cao, Z.; Zhang, Z.; Zhang, Y., Universal transfer of full-class metal electrodes for barrier-free two-dimensional semiconductor contacts. *InfoMat* **2024,** *6* (1), e12491.

7. Liu, G.; Tian, Z.; Yang, Z.; Xue, Z.; Zhang, M.; Hu, X.; Wang, Y.; Yang, Y.; Chu, P. K.; Mei, Y.; Liao, L.; Hu, W.; Di, Z., Graphene-assisted metal transfer printing for wafer-scale integration of metal electrodes and two-dimensional materials. *Nature Electronics* **2022,** *5* (5), 275-280.

8. Kong, L.; Wu, R.; Chen, Y.; Huangfu, Y.; Liu, L.; Li, W.; Lu, D.; Tao, Q.; Song, W.; Li, W.; Lu, Z.; Liu, X.; Li, Y.; Li, Z.; Tong, W.; Ding, S.; Liu, S.; Ma, L.; Ren, L.; Wang, Y.; Liao, L.; Duan, X.; Liu, Y., Wafer-scale and universal van der Waals metal semiconductor contact. *Nature Communications* **2023,** *14* (1), 1014.

9. Yang, X.; Li, J.; Song, R.; Zhao, B.; Tang, J.; Kong, L.; Huang, H.; Zhang, Z.; Liao, L.; Liu, Y.; Duan, X.; Duan, X., Highly reproducible van der Waals integration of two-dimensional electronics on the wafer scale. *Nature Nanotechnology* **2023,** *18* (5), 471-478.

10. Zhang, X.; Huang, C.; Li, Z.; Fu, J.; Tian, J.; Ouyang, Z.; Yang, Y.; Shao, X.; Han, Y.; Qiao, Z.; Zeng, H., Reliable wafer-scale integration of two-dimensional materials and metal electrodes with van der Waals contacts. *Nature Communications* **2024,** *15* (1), 4619.

11. Yu, H.; Liao, M.; Zhao, W.; Liu, G.; Zhou, X. J.; Wei, Z.; Xu, X.; Liu, K.; Hu, Z.; Deng, K.; Zhou, S.; Shi, J.-A.; Gu, L.; Shen, C.; Zhang, T.; Du, L.; Xie, L.; Zhu, J.; Chen, W.; Yang, R.; Shi, D.; Zhang, G., Wafer-Scale Growth and Transfer of Highly-Oriented Monolayer MoS_2 Continuous Films. *ACS Nano* **2017,** *11* (12), 12001-12007.

12. Choi, M.; Park, Y. J.; Sharma, B. K.; Bae, S.-R.; Kim, S. Y.; Ahn, J.-H., Flexible active-matrix organic light-emitting diode display enabled by MoS_2 thin-film transistor. *Science Advances* **2018,** *4* (4), eaas8721.

13. Wang, Y.; Kim, J. C.; Wu, R. J.; Martinez, J.; Song, X.; Yang, J.; Zhao, F.; Mkhoyan, A.; Jeong, H. Y.; Chhowalla, M., Van der Waals contacts between three-dimensional metals and two-dimensional semiconductors. *Nature* **2019,** *568* (7750), 70-74.

14. Sebastian, A.; Pendurthi, R.; Choudhury, T. H.; Redwing, J. M.; Das, S., Benchmarking monolayer MoS_2 and WS_2 field-effect transistors. *Nature Communications* **2021,** *12* (1), 693.

15. Shen, P.-C.; Su, C.; Lin, Y.; Chou, A.-S.; Cheng, C.-C.; Park, J.-H.; Chiu, M.-H.; Lu, A.-Y.; Tang, H.-L.; Tavakoli, M. M.; Pitner, G.; Ji, X.; Cai, Z.; Mao, N.; Wang, J.; Tung, V.; Li, J.; Bokor, J.; Zettl, A.; Wu, C.-I.; Palacios, T.; Li, L.-J.; Kong, J., Ultralow contact resistance between semimetal and monolayer semiconductors. *Nature* **2021,** *593* (7858), 211-217.

16. Mondal, A.; Biswas, C.; Park, S.; Cha, W.; Kang, S.-H.; Yoon, M.; Choi, S. H.; Kim, K. K.; Lee, Y. H., Low Ohmic contact resistance and high on/off ratio in transition metal dichalcogenides field-effect transistors via residue-free transfer. *Nature Nanotechnology* **2024,** *19* (1), 34-43.

17. Kwon, J.; Seol, M.; Yoo, J.; Ryu, H.; Ko, D.-S.; Lee, M.-H.; Lee, E. K.; Yoo, M. S.; Lee, G.-H.; Shin, H.-J.; Kim, J.; Byun, K.-E., 200-mm-wafer-scale integration of polycrystalline molybdenum disulfide transistors. *Nature Electronics* **2024,** *7* (5), 356-364.

18. English, C. D.; Shine, G.; Dorgan, V. E.; Saraswat, K. C.; Pop, E., Improved Contacts to MoS_2 Transistors by Ultra-High Vacuum Metal Deposition. *Nano Letters* **2016,** *16* (6), 3824-3830.

19. Andrews, K.; Bowman, A.; Rijal, U.; Chen, P.-Y.; Zhou, Z., Improved Contacts and Device Performance in MoS_2 Transistors Using a 2D Semiconductor Interlayer. ACS Nano 2020, 14 (5), 6232-6241.

20. Kim, B.-K.; Kim, T.-H.; Choi, D.-H.; Kim, H.; Watanabe, K.; Taniguchi, T.; Rho, H.; Kim, J.-J.; Kim, Y.-H.; Bae, M.-H., Origins of genuine Ohmic van der Waals contact between indium and MoS_2. *npj 2D Materials and Applications* **2021,** *5* (1), 9.

21. Qi, D.; Li, P.; Ou, H.; Wu, D.; Lian, W.; Wang, Z.; Ouyang, F.; Chai, Y.; Zhang, W., Graphene-Enhanced Metal Transfer Printing for Strong van der Waals Contacts between 3D Metals and 2D Semiconductors. *Advanced Functional Materials* **2023,** *33* (27).

22. Li, W.; Gong, X.; Yu, Z.; Ma, L.; Sun, W.; Gao, S.; Köroğlu, Ç.; Wang, W.; Liu, L.; Li, T.; Ning, H.; Fan, D.; Xu, Y.; Tu, X.; Xu, T.; Sun, L.; Wang, W.; Lu, J.; Ni, Z.; Li, J.; Duan, X.; Wang, P.; Nie, Y.; Qiu, H.; Shi, Y.; Pop, E.; Wang, J.; Wang, X., Approaching the quantum limit in two-dimensional semiconductor contacts. *Nature* **2023,** *613* (7943), 274-279.

23. Lu, D.; Chen, Y.; Lu, Z.; Ma, L.; Tao, Q.; Li, Z.; Kong, L.; Liu, L.; Yang, X.; Ding, S.; Liu, X.; Li, Y.; Wu, R.; Wang, Y.; Hu, Y.; Duan, X.; Liao, L.; Liu, Y., Monolithic three-dimensional tier-by-tier integration via van der Waals lamination. *Nature* **2024,** *630* (8016), 340-345.

24. Kim, T.; Mun, J.; Park, H.; Joung, D.; Diware, M.; Won, C.; Park, J.; Jeong, S.-H.; Kang, S.-W., Wafer-scale production of highly uniform two-dimensional MoS_2 by metal-organic chemical vapor deposition. *Nanotechnology* **2017,** *28* (18), 18LT01.

25. Mun, J.; Park, H.; Park, J.; Joung, D.; Lee, S.-K.; Leem, J.; Myoung, J.-M.; Park, J.; Jeong, S.-H.; Chegal, W.; Nam, S.; Kang, S.-W., High-Mobility MoS_2 Directly Grown on Polymer Substrate with Kinetics-Controlled Metal–Organic Chemical Vapor Deposition. *ACS Applied Electronic Materials* **2019,** *1* (4), 608-616.

26. Seol, M.; Lee, M.-H.; Kim, H.; Shin, K. W.; Cho, Y.; Jeon, I.; Jeong, M.; Lee, H.-I.; Park, J.; Shin, H.-J., High-Throughput Growth of Wafer-Scale Monolayer Transition Metal Dichalcogenide via Vertical Ostwald Ripening. *Advanced Materials* **2020,** *32* (42), 2003542.

27. Dodda, A.; Jayachandran, D.; Pannone, A.; Trainor, N.; Stepanoff, S. P.; Steves, M. A.; Radhakrishnan, S. S.; Bachu, S.; Ordonez, C. W.; Shallenberger, J. R.; Redwing, J. M.; Knappenberger, K. L.; Wolfe, D. E.; Das, S., Active pixel sensor matrix based on monolayer MoS_2 phototransistor array. *Nature Materials* **2022,** *21* (12), 1379-1387.

28. Kang, J.-H.; Shin, H.; Kim, K. S.; Song, M.-K.; Lee, D.; Meng, Y.; Choi, C.; Suh, J. M.; Kim, B. J.; Kim, H.; Hoang, A. T.; Park, B.-I.; Zhou, G.; Sundaram, S.; Vuong, P.; Shin, J.; Choe, J.; Xu, Z.; Younas, R.; Kim, J. S.; Han, S.; Lee, S.; Kim, S. O.; Kang, B.; Seo, S.; Ahn, H.; Seo, S.; Reidy, K.; Park, E.; Mun, S.; Park, M.-C.; Lee, S.; Kim, H.-J.; Kum, H. S.; Lin, P.; Hinkle, C.; Ougazzaden, A.; Ahn, J.-H.; Kim, J.; Bae, S.-H., Monolithic 3D integration of 2D materials-based electronics towards ultimate edge computing solutions. *Nature Materials* **2023,** *22* (12), 1470-1477.
